# Dual-Specificity Phosphatase 4 Regulates STAT5 Protein Stability and Helper T Cell Polarization*

**DOI:** 10.1371/journal.pone.0145880

**Published:** 2015-12-28

**Authors:** Wan-Yi Hsiao, Yu-Chun Lin, Fang-Hsuean Liao, Yi-Chiao Chan, Ching-Yu Huang

**Affiliations:** Immunology Research Center, National Health Research Institutes, Zhunan, Miaoli County, Taiwan; University of Lisbon, PORTUGAL

## Abstract

Immune responses are critically regulated by the functions of CD4 helper T cells. Based on their secreted cytokines, helper T cells are further categorized into different subsets like Treg or Th17 cells, which suppress or promote inflammatory responses, respectively. Signals from IL-2 activate the transcription factor STAT5 to promote Treg but suppress Th17 cell differentiation. Our previous results found that the deficiency of a dual-specificity phosphatase, DUSP4, induced STAT5 hyper-activation, enhanced IL-2 signaling, and increased T cell proliferation. In this report, we examined the effects of DUSP4 deficiency on helper T cell differentiation and STAT5 regulation. Our *in vivo* data showed that DUSP4 mice were more resistant to the induction of autoimmune encephalitis, while *in vitro* differentiations revealed enhanced iTreg and reduced Th17 polarization in DUSP4-deficient T cells. To study the cause of this altered helper T cell polarization, we performed luciferase reporter assays and confirmed that, as predicted by our previous report, DUSP4 over-expression suppressed the transcription factor activity of STAT5. Surprisingly, we also found that DUSP4-deficient T but not B cells exhibited elevated STAT5 protein levels, and over-expressed DUSP4 destabilized STAT5 *in vitro*; moreover, this destabilization required the phosphatase activity of DUSP4, and was insensitive to MG132 treatment. Finally, domain-mapping results showed that both the substrate-interacting and the phosphatase domains of DUSP4 were required for its optimal interaction with STAT5, while the coiled-coil domain of STAT5 appeared to hinder this interaction. Our data thus provide the first genetic evidence that DUSP4 is important for helper T cell development. In addition, they also help uncover the novel, DUSP4-mediated regulation of STAT5 protein stability.

## Introduction

Dual-specificity phosphatases (DUSPs) are named for their ability to dephosphorylate both serine/threonine and tyrosine residues of their substrates [1]. Four DUSPs, DUSP1, -2, -4 and -5, share similar structures and nuclear localization [2, 3]. Within these nuclear DUSPs, DUSP4 is reported to dephosphorylate ERK and JNK in cell lines [4, 5], and is often referred to as MAP kinase phosphatase 2/MKP2. Structurally, DUSP4 contains an N-terminal kinase-interacting motif (KIM), a C-terminal phosphatase domain, and two nuclear localization signals that are redundant for transporting DUSP4 into the nucleus [6]. The expression of DUSP4 is tightly controlled both transcriptionally and post-translationally: DUSP4 transcription is rapidly induced by MAPK following mitogenic stimulation [5] or the activation of transcription factors p53 and HoxA10 [7, 8], while DUSP4’s short half-life of ~1/2 hr implies a stringent protein stability regulation [9]. Functionally, DUSP4 has been implicated in playing a role in replicative senescence [9], glucocorticoid response [10], and oxidative stress response [11]. The analyses of DUSP4-deficient mice further show that DUSP4 is important for TGFβ-induced apoptosis [12], inflammatory cytokine secretion, susceptibility to sepsis shock [13], and resistance to *Leishmania mexicana* infection [14]. Finally, our previous results show that DUSP4 dephosphorylates STAT5 to negatively regulate IL-2 signaling and CD4 T cell proliferation [15], while a recent report suggests that age-related DUSP4 expression impairs T-dependent B cell responses [16].

CD4 helper T (Th) cells normally polarize into different subsets such as Th1, Th2, Th17 and Treg, with each subset carrying unique distributions and immune regulatory functions. This polarization process is controlled by cytokine signaling, and is mediated through the functions of “master transcription factors” [17]. In this regard, IL-2 is essential for the differentiation and maintenance of Treg cells that are governed by the transcriptional repressor FOXP3 [18, 19], while Th17 differentiation, overseen by Rorγt/Rorα, is inhibited by IL-2 [20]. These various Th subsets play important roles in mediating the protective immunity for the host, such that Th1 promotes inflammation, Th2 facilitates the production of antibodies, Treg suppresses unwanted immune responses, and Th17 conveys protections from fungi and bacteria [17]. However, hinging on their broad immune-regulatory functions, imbalanced Th cell activities are considered to be the source of many autoimmune disorders [17]. Along this line, Treg, Th17, and, more recently, Th1 cells have been shown to participate in the pathogenesis of neuron demyelination in experimental autoimmune encephalitis (EAE), a mouse model for human multiple sclerosis [21–23].

Prior to its identification as a helper T cell polarization factor, IL-2 is reported to function through autocrine and paracrine to amplify and sustain the proliferation of T cells following their activations [24]. Resting T cells express the intermediate-affinity IL-2 receptor (IL-2R) IL-2Rβγ complex. After IL-2 activates the IL-2Rβγ complex and subsequently JAK1/JAK3 tyrosine kinases, the transcription factor STAT5 is phosphorylated, dimerized, and transported into the nucleus. Recent studies suggest that phosphorylated STATs forms a parallel dimer via a trans-interaction between the Y694 residue and the c-terminal SH2 domain [25]; this specific dimer configuration then facilitates the nuclear import and DNA-binding of STAT5 so that down-stream transcriptional activations can occur (reviewed in [26, 27]). Together with other transcription factors such as NFAT and NFκB, STAT5 induces the expression of CD25, or IL-2Rα subunit, to form the high-affinity IL-2Rαβγ and further amplify IL-2 signaling through positive feedback regulations (reviewed in [24]). In addition to IL-2, STAT5 can also be activated by IL-7, GM-CSF [28], and a cohort of other cytokines [29] to induce a broad spectrum of genes involved in immune regulations. Recent reports show that STAT5 is required for the optimal induction [30] or homeostasis [31] of Treg cells. Moreover, STAT5 can also crosstalk with STAT3 to coordinate the polarization of Treg and Th17 cells (reviewed in [29]). The regulation of STAT5 is thus pivotal for mediating proper helper T cells responses.

The activation of STAT5 depends on the phosphorylation of Y694, as Y694F mutations abrogate its transcription activity [32]. Several serine residues are also phosphorylated on activated STAT5 [33, 34], but they do not appear to be essential for IL-2-mediated T cell proliferation [34]. While the phosphorylation and activation of STAT5 by JAK1 and JAK3 kinases is clear, the mechanism for inactivating STAT5 is not completely understood. Two phosphatases, DUSP4 [15] and SHP-2 [35], have been reported to dephosphorylate STAT5 and down-regulate its transcription activity; yet several other phosphatases, including SHP-1, DUSP3/VHR and PTP1B, have also been implicated [36–38]. In addition, the activity of STAT5 can likewise be regulated by its nuclear shuttling [39, 40] or degradation [41–43]; however, the mediators behind these regulations remain elusive.

In this report, we extended from our previous study [15] to further investigate how DUSP4 may regulate the activity of STAT5. By charactering primary DUSP4^-/-^ T cells and HEK-293 cells with exogenous expression of DUSP4, our analyses showed that DUSP4, besides dephosphorylating STAT5, may also down-regulate STAT5 to modulate its activity. Furthermore, we also investigated the *in vivo* consequences of STAT5 hyper-activation in DUSP4^-/-^ T cells in the context of helper T cell differentiation and autoimmune regulation. Our results suggest that Treg/Th17 polarization and susceptible to EAE induction were both regulated by DUSP4.

## Materials and Methods

### Ethics statement

No human subject was involved in this study. The use of animals was carried out according to the guidelines set forth by the Institutional Animal Care and Use Committee of the National Health Research Institutes under the approved protocol numbers NHRI-IACUC-100147-A and NHRI-IACUC-103112-A Mice were housed in the specific-pathogen-free facility at the National Health Research Institutes. Cares were taken to provide optimal housing for the animals, and euthanasia was practiced to minimize the pain of the animals in all experiments.

### Mice

FOXP3-GFP (kindly provided by Dr. Alexander Rudensky at the Memorial Sloan Kettering Cancer Center) [44] and DUSP4^-/-^ [15] mice have been described. DUSP4^-/-^:FOXP3-GFP^+^ mice were generated by cross-breeding. C57Bl/6 mice were obtained from the National Laboratory Animal Center, Taiwan. All mice were back-crossed to C57Bl/6 mice for more than ten generations prior to analyses. Animals were housed in the specific pathogen-free facility at the Laboratory Animal Center of the National Health Research Institutes (NHRI). All animal experimental procedures were preapproved by the NHRI’s Institutional Animal Care and Use Committee.

### Genotyping PCR, two-template PCR and quantitative-PCR (qPCR)

Genotyping PCR for the DUSP4 knockout and FOXP3-GFP alleles was performed using extracted tail DNA with Phire polymerase (Thermo Scientific). PCR for generating the WT or mutant DUSP4 and STAT5 constructs, as well as ametrine, GFP and tdTomato inserts, was performed with Phusion high-fidelity polymerase (Thermo Scientific) according to the supplier’s suggestions; for secondary PCR in two-template PCR, 1 ng of primary PCR products extracted with illustra Gel Purification kit (GE Healthcare) was used as the template. All PCR reactions were carried out on Mastercycler S (Eppendorf). Phire PCR cycling conditions were: initial denaturing at 98°C for 2 m, followed by 28 cycles of denaturing temperature at 98°C for 30 s, annealing temperature at 62°C for 30 s, and extension temperature at 72°C for 30 s; the program then ended with a single step of 72°C for 5 m. Phusion PCR cycling conditions were: initial denaturing at 98°C for 2 m, followed by various cycles (S1 Table) of denaturing temperature at 98°C for 30 s, primer-specific annealing temperature (S1 Table) for 30 s, and extension temperature at 72°C for 30 s; the program then ended with a single step of 72°C for 5 m. For RT-qPCR, RNA was extracted with Trizol (Invitrogen) and cDNA was synthesized with First Strand cDNA Synthesis Kit (Thermo Scientific) following the manufacturers’ protocol. qPCR reactions were performed with Luminaris Color HiGreen High ROX qPCR Master Mix (Thermo Scientific) on Realplex4 with Mastercycler ep realplex software (Eppendorf). qPCR PCR cycling conditions were: initial denaturing at 95°C for 10 m, followed by 40 cycles of denaturing temperature at 95°C for 20 s, annealing temperature at 55°C for 30 s, and extension temperature at 72°C for 30 s. All primer sequences, annealing temperatures, and cycle numbers can be found in S1 Table.

### Plasmids constructions and transfections

Mouse STAT5a and DUSP4 coding sequences were amplified from C57Bl/6 splenic cDNA and cloned into the TrueORF pCMV6-AC-DDK/Flag or pCMV6-AC-Myc vectors (OriGene) between the AsiSI and MluI sites. Alternative STAT5a cDNA constructs were kindly provided by Dr. Toshio Kitamura at University of Tokyo. All truncation, deletion, or point-mutation STAT5a and DUSP4 constructs, except for the STAT5-SH2 mutant, were generated via regular or two-template PCR and cloned into the respective vectors. The STAT5-SH2 deletion mutant was generated by amplifying separately the 5’ (AsiSI+EcoRV) and 3’ (EcoRV+MluI) fragments with RT-PCR, followed by a three-party ligation into AsiSI and MluI digested TrueORF pCMV6-AC-DDK. The vector containing the ametrine fluorescent protein was kindly provided by Dr. Robert E. Campbell at the University of Alberta [45] via AddGene (plasmid 18879), and was used for PCR amplification of the ametrine sequences (without the STOP codon) for cloning into the AsiSI site of pCMV6-AC-DDK-STAT5 to generate the ametrine-STAT5 fusion construct. The GFP-STAT5 fusion construct and the control tdTomato construct were similarly generated by PCR. For generating the Tet-on DUSP4 constructs, WT DUSP4 and phosphatase-dead (PD) DUSP4 sequences were lifted from the pCMV6-AC-Myc backbone via BamHI and XhoI, and ligated into BamHI+XhoI-cut pcDNA4-TO-myc-His A supplied in the T-REx system (Invitrogen). The STAT5 LHRE luciferase reporter plasmid was kindly provided by Dr. Richard J. Ross at the University of Sheffield [46]. All generated constructs were validated by sequencing. Transient and stable transfections of HEK-293/293T cells were performed with polyethylenimine (Sigma-Aldrich) following standard protocols.

### Flow cytometry

Antibodies used for flow cytometry, including CD4, CD8 and IL-17A conjugated with various fluorescent dyes, were purchased (BioLegend or BD Biosciences). Cells were stained with specific antibodies on ice for 10 m in DMEM supplemented with 2% fetal bovine serum (FBS) (BioLegend) and 0.1% NaN_3_ (Sigma-Aldrich), followed by washing and resuspending in 1X PBS with 0.1% NaN_3_ for analyses. Intracellular staining was performed with Cytofix/Cytoperm buffers (BD Biosciences) following the manufacturer’s condition. Flow cytometry results were captured on FACSCanto II (BD Biosciences), with the data analyzed by the FlowJo software (FlowJo). All FACS gating strategies and sorting results are shown in Figure A-C in S1 Fig.

### Cell sorting, culture, and the generation of tetracycline-inducible clones

Naïve CD4 T cells were purified (to ~90–95% CD4^+^ purity, within which ~80% were CD62L^+^ naïve cells and ~7% were FOXP3-GFP^+^ nTreg cells; see Figure C in S1 Fig for post-sort results) via negative selection of B220^+^, CD11b^+^, CD49b^+^, Ter119^+^, and CD8^+^ cells from RBC-lysed splenocytes using magnetic-assisted cell sorting (MACS) kits (Miltenyi Biotec) with standard protocols. Purified CD4 T cells were cultured in DMEM supplemented with 1x non-essential amino acid, 2 mM L-glutamine, 2 mM Glutamax, 1 mM sodium pyruvate, 10 mM HEPES (Invitrogen), 10% FBS, 100 U/ml penicillin, 100 mg/ml streptomycin (Biological Industries), and 125 mM 2-ME (Sigma-Aldrich). iTreg and iTh17 polarizations were performed by stimulating purified naïve CD4 T cells with 1.6 μg/ml plate-bound anti-CD3 and 1.6 μg/ml soluble anti-CD28 (BioLegend), in the presence of 5 ng/ml TGFβ (PeproTech) + 10 μg/ml anti-IL-4 (BioLegend) + 10μ g/ml anti-IFNγ (BioLegend), or 30 ng/ml IL-6 (PeproTech) + 1 ng/ml TGFβ + 10 μg/ml anti-IFNγ + 10 μg/ml anti-IL-4, respectively. HEK-293 and -293T cells were cultured in DMEM with 10% FBS, 100 U/ml penicillin and 100 mg/ml streptomycin. Tetracycline-inducible HEK-293 clones were generated by transfecting HEK-293 cells with pcDNA6-TR from the T-Rex system (Invitrogen), followed by selection in 4 μg/ml blasticidin (Invitrogen) for 7 d and subcloning by limiting dilution. Blasticidin-resistant clones were tested for tetracycline-inducibility by transient transfections of the Tet-on GFP plasmid and overnight culture with or without 2–10 μg/ml tetracycline (Sigma-Aldrich) or doxycycline (Sigma-Aldrich), followed by flow cytometry analyses for GFP induction. The tetracycline-inducible clones were then transfected with the respective Tet-on constructs, followed by selection and subcloning in the presence of 400 μg/ml Zeocin (Invitrogen). The induction of Tet-on gene expression was performed with 2–10 μg/ml tetracycline or doxycycline. For generating the 293-TO-D4-WT lines with stable expression of the GFP-STAT5 fusion reporter, 293-TO-D4-WT cells were transfected with linearized GFP-STAT5 constructs and repetitively sorted for GFP^+^ cells until GFP expression stabilized ~3 month later. Unless otherwise indicated, hIFNβ was used at 30 ng/ml, proteasome inhibitor MG132 (Calbiochem) was used at 5 μM, protein synthesis inhibitor cycloheximide (Enzo Life Sciences) was used at 100 μg/ml, and lysosome inhibitor chloroquine (Sigma-Aldrich) was used as 25 μM.

### Luciferase reporter assays

HEK-293T or tetracycline-inducible 293-TO-D4-WT/293-TO-D4-PD cells were transiently transfected with the STAT5 LHRE luciferase reporter [46], WT-STAT5, and a control tdTomato plasmid using Polyethylenimine (Sigma-Aldrich) with standard protocols; cells were either co-transfected with DUSP4 constructs, or cultured in the presence of 2 μg/ml tetracycline or doxycycline overnight to induce DUSP4 expression. On the following day, 50 ng/ml human IFNβ (hIFNβ) (PeproTech) was added to the culture to activate STAT5-mediated luciferase transcription overnight. The luciferase activity was measured on the next day using the Neolite luciferase reporter assay system (Perkin-Elmer) on Luminometer TD 20/20 (Turner Designs) following the manufacturer’s protocol. A small fraction of the transfected cells was subjected to flow cytometry analyses for the percentages of tdTomato^+^ cells as an index of transfection efficiency for normalizing the luciferase signals.

### Western blotting

Antibodies against STAT5 (Cell Signaling), phospho-STAT5 (Tyr-694) (Cell Signaling), and β-tubulin (GeneTex) were purchased. Polyclonal rabbit antibodies against DUSP4 were generated by peptide immunization (YDERSPRAESLREDSTV-C). Lysate preparation, western blotting, antibody hybridization and chemiluminescent reactions were performed with standard protocols. Western blots were visualized and quantified on BioSpectrum Imagining System with Vision-Works LS software (UVP) or with the ImageJ software (ImageJ).

### Immunoprecipitation (IP)

HEK-293T cells were transfected with STAT5 and DUSP4 plasmids and cultured overnight. After incubating in 10 ng/ml hIFNβ and 25 μM pervanadate (Sigma-Aldrich) for 30 m, cells were washed with cold 1X PBS and lysed in SDS-free RIPA buffer (50 mM Tris-HCl, 0.25% sodium deoxycholate, 150 mM NaCl, 0.1% NP-40, 1 mM EDTA) on ice for 30 m. Lysates were pre-cleared by incubating with agarose-G beads (Millipore) at 4°C on a rotating platform for 30 m. A small fraction of the pre-cleared lysates was saved as pre-IP samples, while ~1 mg pre-cleared lysates were incubated with the Anti-Flag M2 affinity gel (Sigma-Aldrich) at 4°C on a rotating platform for 3 h, followed by three 1 m washes with SDS-free RIPA buffer and 10000g centrifugation. The washed gel beads were then added with sample buffer (5X buffer: 250 mM Tris pH6.8, 50% glycerol, 10% SDS, with bromophenol blue) to 1X, boiled for 3 m, and centrifuged at 10000g for 1 m. Supernatants were then collected and analyzed alongside the pre-IP samples by western blotting.

### EAE induction

Mice were immunized with 0.033 ml emulsion of myelin oligodendrocyte glycoprotein 35–55 peptide (1 mg/ml) in complete Freund’s adjuvant (Difco) on three sites (one on midline back and two on lower side-back), as well as two injections of 200 ng pertussis toxin (Sigma-Aldrich) i.p. (one on the same day and one 2 d later). Mice were then monitored daily for signs of encephalitis (0: normal; 1: limp tail or hind limb weakness but not both; 2: limp tail and hind limb weakness; 3 partial hind limb paralysis; 4: complete hind limb paralysis; 5: moribund). It should be noted that no moribund state was reached in our analyses.

### Statistical analyses

In all assays, data were plotted as mean±SEM, with the *p*-values calculated with unpaired or paired two-tailed Student’s *t*-test, or with one-sample *t*-test when the samples were compared with normalized WT controls with a hypothetical value of 1. All statistical analyses were performed by GraphPad Prism software (GraphPad Software). *p*-values larger than 0.05 were either unlabeled or were shown in parenthesis.

## Results

### DUSP4 deficiency enhances resistance to EAE, and alters Th17 and Treg development

**Fig 1:**
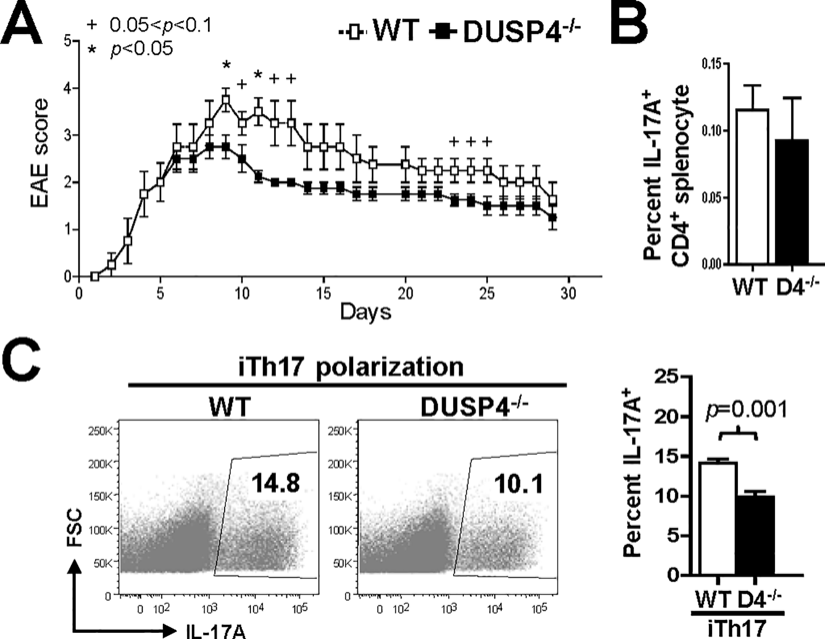
DUSP4 deficiency confers resistance to EAE induction and alters Th17 polarization. (A) Mice of 9–12 wk age were immunized with MOG peptide and diphtheria toxin to induce EAE. The disease scores were recorded daily (*n =* 4). Asterisk, *p*<0.05 between WT and DUSP4^-/-^ mice for the given time point. Plus sign, 0.05<*p*<0.1 between WT and DUSP4^-/-^ mice for the given time point. (B) Splenocytes from 6–12 wk old mice were analyzed by flow cytometry. The percentages of intracellular IL-17A^+^ population in CD4^+^ T cells are shown (*n* = 3). D4^-/-^, DUSP4^-/-^. (C) Naïve CD4 T cells were purified and cultured in iTh17-polarizing conditions for 3 d, followed by flow cytometry analyses. Representative FACS plots (left panels) and statistical analyses results of the percentages of intracellular IL-17A^+^ cells in CD4^+^ populations (right panel) are shown (*n =* 5–6).

Our previous report shows that IL-2 signaling is enhanced in DUSP4-deficient mice [15]. Since the development of Treg cells is positively regulated by IL-2 [18, 19], while exogenous IL-2 suppresses the polarization of Th17 cell [20], we then set out to determine whether the altered IL-2 responses in DUSP4 deficient mice also affected autoimmune responses associated with imbalanced Th development. We first subjected WT and DUSP4^-/-^ mice to the induction of EAE, an autoimmune mouse model of human multiple sclerosis, in which the symptoms are ameliorated by Treg but aggravated by Th17 cells [21–23]. The results indicated that DUSP4^-/-^ mice were associated with enhanced resistance to EAE induction (Fig 1A). The analyses of primary splenocytes showed that the numbers of Th17 cells were only slightly reduced in DUSP4^-/-^ mice (Fig 1B); however, the efficiency of *in vitro* Th17 polarization (iTh17) was significantly reduced in DUSP4^-/-^ CD4 T cells (*p =* 0.001, Fig 1C).

**Fig 2:**
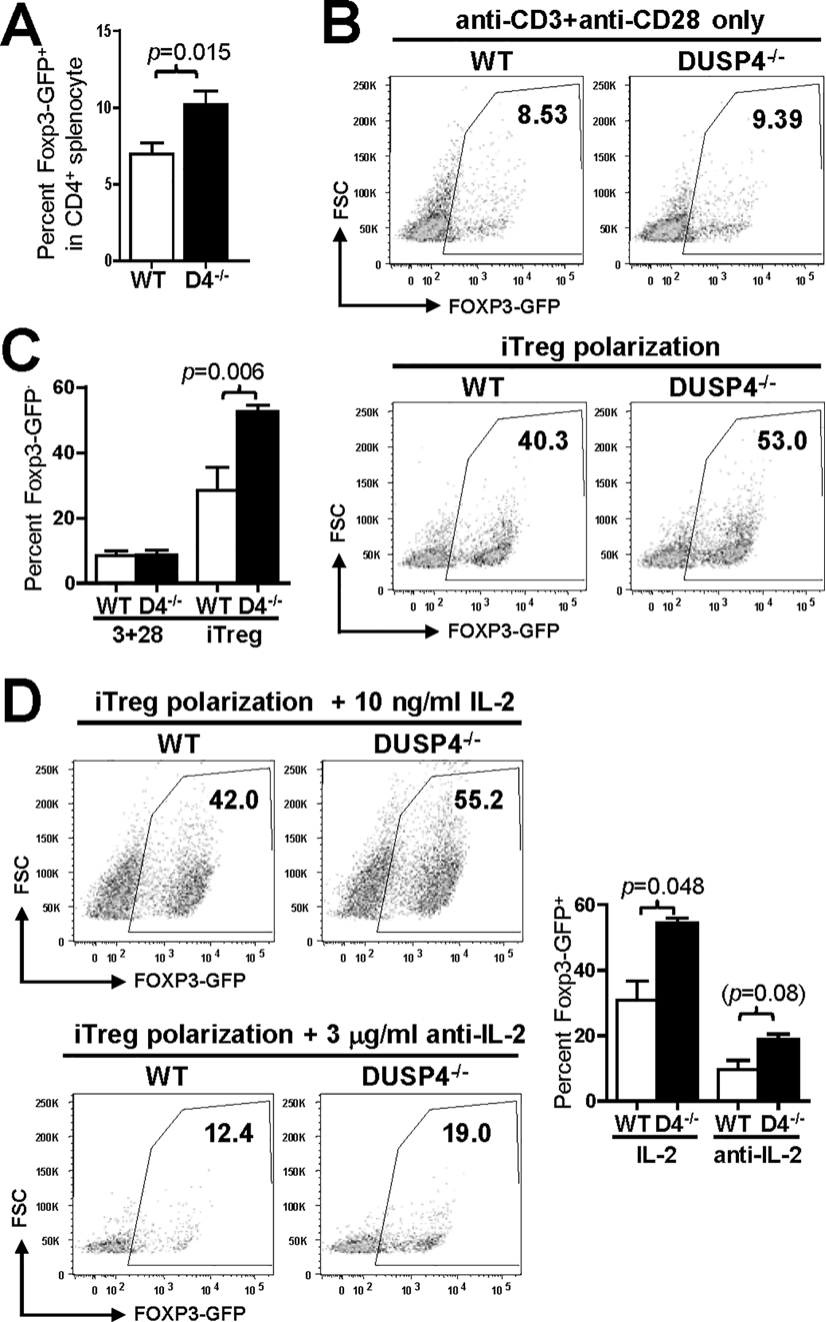
DUSP4 deficiency enhances Treg homeostasis and iTreg polarization. (A) Splenocytes from 6–12 wk old FOXP3-GFP^+^ or DUS4^-/-^:FOXP3-GFP^+^ mice were analyzed by flow cytometry. The percentages of FOXP3-GFP^+^ population in CD4^+^ T cells are shown (*n =* 8–11). D4^-/-^, DUSP4^-/-^. (B-C) Purified naïve CD4 T cells from FOXP3-GFP^+^ mice were cultured with anti-CD3ε+anti-CD28 only (3+28) or under iTreg polarizing conditions (iTreg) for 3 d and analyzed by flow cytometry. Representative FACS plots (panel B) and statistical analyses results of the percentages of FOXP3-GFP^+^ cells in CD4^+^ populations (panel C) are shown (*n =* 3–5). (D) Purified naïve CD4 T cells from FOXP3-GFP^+^ mice were induced for iTreg as in panel B, except with the addition of exogenous IL-2 or IL-2-neutralizing antibodies as indicated. Representative FACS plots (left panels) and statistical analyses results of the percentages of FOXP3-GFP^+^ cells in CD4^+^ T cells (right panel) are shown (*n =* 3).

In contrast to the small reduction of Th17 *in vivo* (Fig 1B), the splenic Treg population was significantly increased in DUSP4^-/-^ mice (*p* = 0.015, Fig 2A). This result differs from our previous observations [15], and may reflect the difference in the genetic background of DUSP4^-/-^ mice (mixed 129/C57Bl/6 background in the previous report, and clean C57Bl/6 background in this report). Moreover, under Treg-polarizing (iTreg) conditions, DUSP4-deficient CD4 T cells were significantly more efficient than their WT counterparts for the differentiation into FOXP3-GFP^+^ Treg cells (*p* = 0.006, Fig 2B and 2C); under neutral conditions (anti-CD3 and anti-CD28 only), the basal induction of FOXP3-GFP was not affected by DUSP4 deficiency (Fig 2B and 2C). To test whether the altered iTreg polarization was mediated solely through enhanced IL-2 production in DUSP4^-/-^ T cells [15], iTreg polarization was induced in the presence of exogenous IL-2 or IL-2 neutralizing antibodies. The results showed that the addition of exogenous IL-2 failed to nullify the advantages of DUSP4^-/-^ CD4 T cells for Treg differentiation (*p =* 0.048, Fig 2D). In contrast, neutralizing antibodies for IL-2 reduced the efficacy of Treg differentiation in both WT and DUSP4^-/-^ T cells and diminished the difference between WT and DUSP4^-/-^ T cells (*p =* 0.08, Fig 2D). The reduced iTh17 and enhanced iTreg polarization of DUSP4^-/-^ CD4 T cells thus link DUSP4 deficiency with altered Th cell development, and provide a mechanistic explanation for the enhanced resistance to autoimmune encephalitis; results from the exogenous IL-2 and anti-IL-2 antibody treatments further suggest IL-2 signaling, but not IL-2 secretion *per se*, are likely involved in this scenario.

### The transcription factor activity and protein stability of STAT5 are both negatively regulated by DUSP4

**Fig 3:**
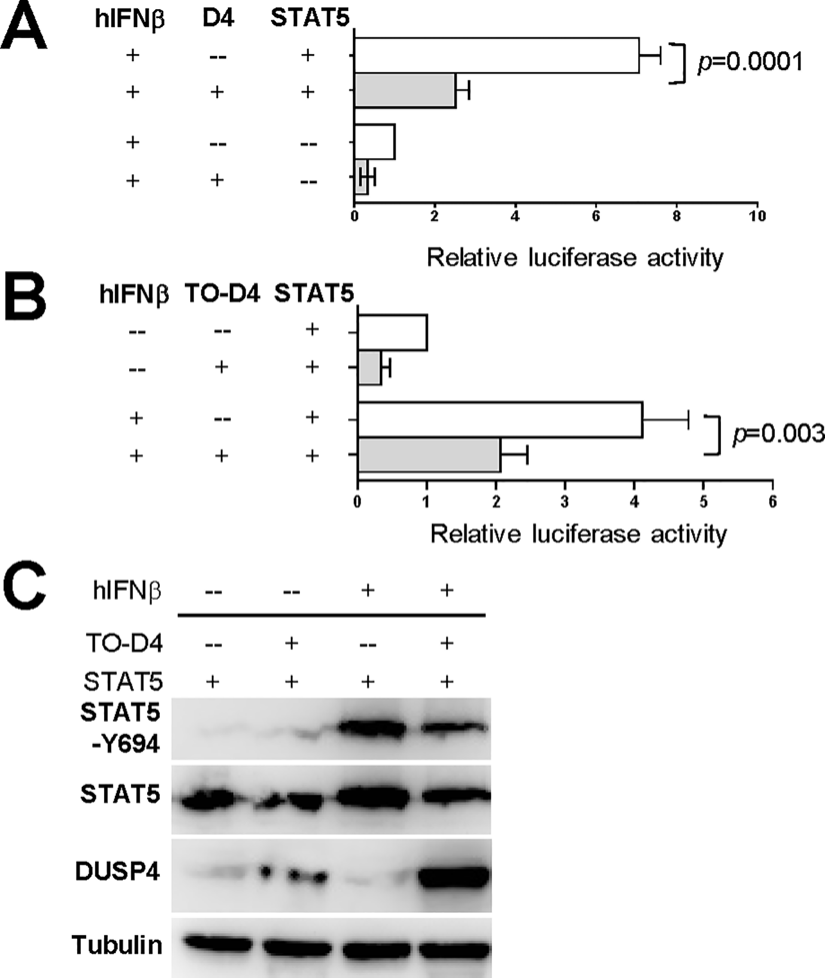
DUSP4 over-expression reduces the transcription factor activity of STAT5. (A) HEK-293T cells were co-transfection with the luciferase reporter, STAT5, DUSP4, and tdTomato as indicated (total DNA amounts across samples were equalized with empty vectors) and cultured for 24 hr, followed by hIFNβ treatment for another 24 hr. Relative luciferase activities were calculated by dividing the luciferase readout with the percentages of tdTomato^+^ cells to compensate for variations in transfection efficiency, followed by normalizing the ratios to untreated WT samples. Results from three independent clones are pooled (*n =* 3). D4, DUSP4. (B) 293-TO-D4-WT clones were transfected with STAT5 and control tdTomato plasmids, treated with tetracycline, and cultured for 24 hr, followed by stimulation with hIFNβ for 24 hr prior to luciferase activity measurement as in panel A. Results from three independent clones are pooled (*n =* 5–6). TO-D4, tetracycline-induced WT DUSP4. (C) Cells were transfected and treated as in B, and were subjected to western blotting analyses. Representative results from three experiments are shown.

**Fig 4:**
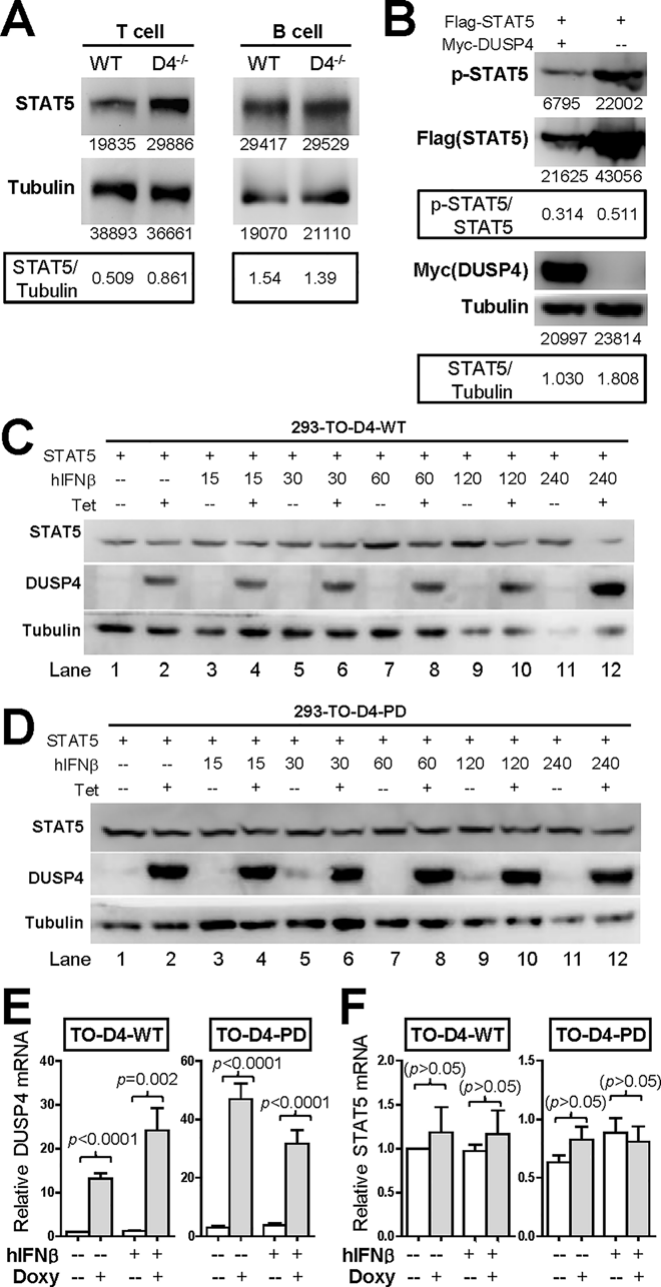
DUSP4 negatively regulates the steady-state levels of STAT5 via its phosphatase activity. (A) MACS-sorted splenic T or B cells were subjected to western blotting analyses. The respective band intensities as well as the STAT5/Tubulin signal ratios are shown. Representative blotting results from four experiments are shown. See also Figure A in S2 Fig. (B) STAT5 and DUSP4 were transiently expressed in HEK-293T cells for 24 hr. Cell lysates were harvested and analyzed by western blotting. The respective band intensities as well as the p-STAT5/STAT5 and STAT5/Tubulin signal ratios are shown. Representative blotting results from three experiments are shown. See also Figure B in S2 Fig p-STAT5, Y694-phosphorylated STAT5. (C) 293-TO-D4-WT clones were transiently transfected with STAT5 and treated with tetracycline for 24 hr. Individual wells were treated with hIFNβ for the indicated time, with all wells harvested simultaneously for western blotting analyses. Representative blotting results from three experiments are shown. Tet, tetracycline. (D) 293-TO-D4-PD clones were similarly transfected with STAT5 and analyzed as in panel C. (E-F). 293-TO-D4-WT (TO-D4-WT) and 293-TO-D4-PD (TO-D4-PD) clones were transfected with WT STAT5 and treated as in panel C except with fixed 4 hr hIFNβ stimulation at the end of the culture period. This was followed by RNA extraction, cDNA synthesis and qPCR analyses to quantify the levels of DUSP4 (panel E) and STAT5 (panel F) mRNA. Relative mRNA levels after compensating for β-actin signals and normalizing to the untreated 293-TO-D4-WT control are shown. Results from two or three independent experiments are pooled (triplicated). Doxy, doxycycline.

STAT5, downstream of the IL-2 signaling cascade, was reported to be dephosphorylated by DUSP4 [15]. To test whether this dephosphorylation translates into altered STAT5 transcription activity, we performed STAT5 luciferase reporter assays in the presence of either transiently-transduced or tetracycline/doxycycline-inducible (Tet-on) DUSP4. The results showed that the human IFNβ (hIFNβ)-induced STAT5 luciferase reporter activity in HEK-293T cells [47] was significantly suppressed by the co-transfection of DUSP4 when compared with vector only controls (*p =* 0.0001, Fig 3A). Similarly, the addition of tetracycline and the resulting induction of DUSP4 in HEK-293 Tet-on (293-TO-D4-WT) cells also significantly suppressed the luciferase activity from the STAT5 reporter (*p =* 0.003, Fig 3B); hIFNβ-induced STAT5 Y294 phosphorylation and Tet-on DUSP4 expression were validated by western blotting (Fig 3C). These data confirm, as predicted by the DUSP4-mediation STAT5 dephosphorylation in our previous report [15], that DUSP4 negatively regulates the transcription activity of STAT5. Interestingly, when examining DUSP4^-/-^ T cell via western blotting, we found that the steady-state level of STAT5 was increased in DUSP4-deficient T, but not B, cells (Fig 4A). Conversely, the over-expression of DUSP4 reduced the level of STAT5 proteins in HEK-293T cells in transient transfection assays i*n vitro* (Fig 4B); meanwhile, tetracycline-induced WT DUSP4 (293-TO-D4-WT, Fig 4C) but not phosphatase-dead (PD) mutant DUSP4 (293-TO-D4-PD, C284S mutation, Fig 4D) also reduced the level of STAT5 proteins in Tet-on clones. Lastly, qPCR analyses confirmed the induction of DUSP4 mRNA in tetracycline-treated Tet-on clones (Fig 4E) and, most importantly, demonstrated that STAT5 mRNA levels were not significantly altered by DUSP4 over-expression (Fig 4F). Similar STAT5a/STAT5b expression levels were also demonstrated in primary WT and DUSP4^-/-^ T cells in microarray analyses (Figure A in S3 Fig). These observations then raise the possibility that, in addition to dephosphorylating STAT5 [15], DUSP4 may also regulate the activity of STAT5 via post-transcriptional modulation of STAT5 protein stability.

**Fig 5:**
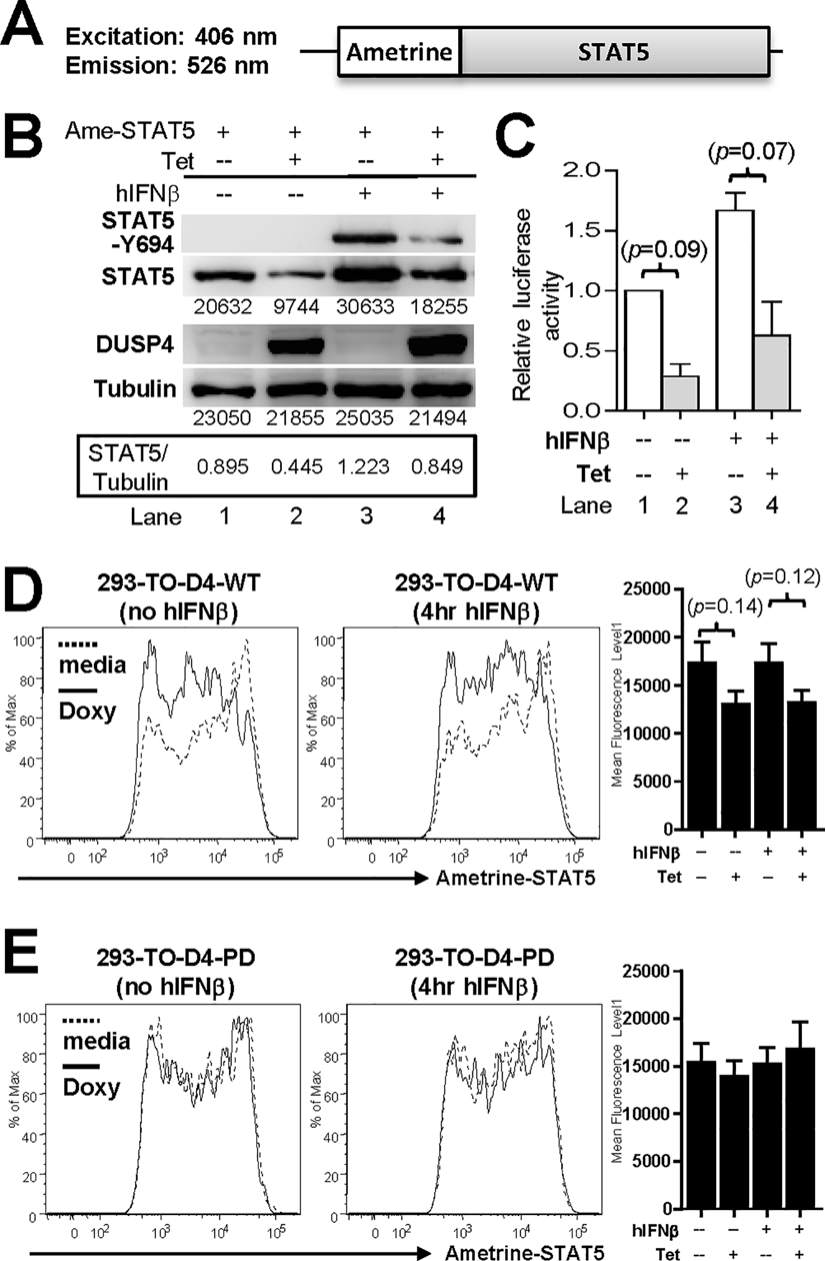
Ametrine-STAT5 fusion protein retains the characteristics of WT STAT5. (A) Schematics of the ametrine-STAT5 fusion protein. Excitation and emission wave-lengths for the ametrine fluorescent protein are also shown. (B) 293-TO-D4-WT clones were transfected with ametrine-STAT5 and treated with tetracycline as indicated for 24 hr, followed by stimulation with hIFNβ for 4 hr prior to lysate collection and western blotting analyses. The respective band intensities as well as the STAT5/Tubulin signal ratios are indicated. Representative results from three experiments are shown. Ame-STAT5, ametrine-STAT5. Tet, tetracycline. (C) 293-TO-D4-WT cells were transfected and treated as in Fig 3A with the addition of luciferase reporter and tdTomato control vectors, and were then analyzed by luciferase reporter assays (*n =* 2–3). (D) 293-TO-D4-WT cells were transfected with ametrine-STAT5 and treated with doxycycline for 24 hr, followed by 4 hr culture with (right panel) or without (left panel) hIFNβ prior to flow cytometry analyses of ametrine-STAT5 levels. Histogram overlays of gated ametrine^+^ cells as well as statistical analyses results of the mean fluorescence levels (right panel) from three independent clones are shown (n = 5). Doxy, doxycycline. (E) 293-TO-D4-PD cells were treated and analyzed as in panel D using three independent clones (n = 5).

To further test this hypothesis, we generated a chimeric ametrine-STAT5 fusion construct (Fig 5A) so that the levels of STAT5 can be directly monitored via the fluorescence intensity of ametrine by flow cytometry. However, prior to testing the effects of DUSP4 on the chimeric protein, we wish to ensure that the prefix of ametrine does not impair the functionality of STAT5. This was supported by western blotting results showing that hIFNβ could induce Y694 phosphorylation of ametrine-STAT5 (compare lane 1 and 3, Fig 5B), and by luciferase reporter assays showing that ametrine-STAT5 could mediate hIFNβ-induced luciferase transcription in 293-TO-D4-WT cells (*p* = 0.09, compare lane 1 and 3, Fig 5C; albeit the induction was slightly less that of WT STAT5 in Fig 3B). Furthermore, this luciferase reporter activity was also slightly reduced by DUSP4 expression (*p* = 0.07, compare lane 3 and 4, Fig 5C) to recapitulate the results from WT STAT5 (Fig 3A and 3B). Finally, ametrine-STAT5 showed similar susceptibilities as WT STAT5 to the DUSP4-mediated protein level modulation (latter results). The ametrine-STAT5 fusion protein thus functions similarly to WT STAT5, and can be used to assess the effects of DUSP4 over-expression on STAT5 protein stability.

Indeed, when DUSP4 was induced by tetracycline, the levels of ametrine-STAT5 fusion protein were decreased in the presence or absence of hIFNβ (compare lane 1 and 2, or lane 3 and 4, Fig 5B). To validate these western blotting results, the same experiments were performed except that the levels of ametrine-STAT5 were monitored by flow cytometry. Flow cytometry analyses on gated ametrine-STAT5^+^ cells showed that the mean fluorescence levels of ametrine were reduced when WT DUSP4 was induced by doxycycline (Fig 5D, left panel)(here doxycycline was chosen over tetracycline due to its lower background fluorescence); the addition of hIFNβ did not significantly alter this reduction (Fig 5D, right panel). That the *p* value failed to reach statistical significance might be attributed to inter-experiment variations resulting from transient ametrine-STAT5 transfections or clonal differences. In contrast, when the same experiments were performed using three independent 293-TO-D4-PD clones, no change in ametrine-STAT5 levels was observed (Fig 5E). Combined with the previous western blotting results (Figs 4C, 4D and 5B), these data suggest that DUSP4 is indeed a negative regulator for the steady-state levels of STAT5 proteins; additionally, this negative regulation requires DUSP4’s phosphatase activity.

### DUSP4 mediates the destabilization of STAT5 via proteasome- and lysosome-independent pathways

**Fig 6:**
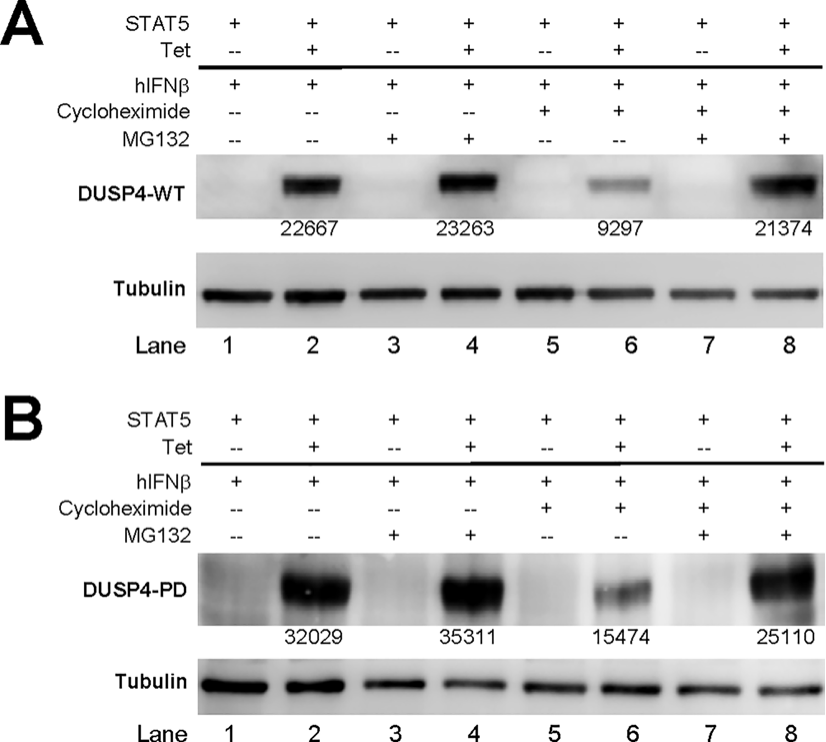
Cycloheximide effectively reduces the level of DUSP4 proteins, while MG132 rescue this reduction. (A) 293-TO-D4-WT clones were transfected, treated, and analyzed by western blotting as in Fig 4C, except with the addition of cycloheximide and MG132 as indicated during the last 6 hr culture period. The respective band intensities are indicated. Representative results from four experiments are shown. (B) 293-TO-D4-PD clones were transfected and analyzed as in panel A. The respective band intensities are indicated. Representative results from three experiments are shown.

Interested in defining the mechanisms for this negative regulation, we resorted to inhibitors of various protein homeostasis pathways, such as cycloheximide (protein translation), MG132 (proteasome-mediated degradation) and chloroquine (lysosome-mediated degradation). However, our initial analyses found that cycloheximide treatment severely reduced the protein level of both WT-DUSP4 (comparing lane 2 and 6, Fig 6A) and PD-DUSP4 (comparing lane 2 and 6, Fig 6B) in Tet-on clones. Meanwhile, MG132 treatment had no significant effect in the absence of cycloheximide (comparing lane 2 and 4, Fig 6A and 6B), but rescued the levels of DUSP4 when cycloheximide was added (comparing lane 6 and 8, Fig 6A and 6B). These observations are in agreement with the reported short half-life of DUSP4 [9], and suggest that DUSP4 protein stability is tightly regulated. More importantly, they also imply that cycloheximide cannot be used to assess the effects of DUSP4 on STAT5 degradation, as cycloheximide may simultaneously reduce (by suppressing STAT5 translation) and enhance (by suppressing DUSP4 translation) the protein level of STAT5. As such, all subsequent assays were performed in the absence of cycloheximide. In addition, DUSP4 Tet-on cells with stable transfections of STAT5 were also generated to bypass potential issues that might arise from the dynamic nature of transient STAT5 transfection. Lastly, GFP-STAT5, instead of ametrine-STAT5 fusion protein, was used to minimize fluorescence spectrum overlaps with tetracycline and doxycycline.

**Fig 7:**
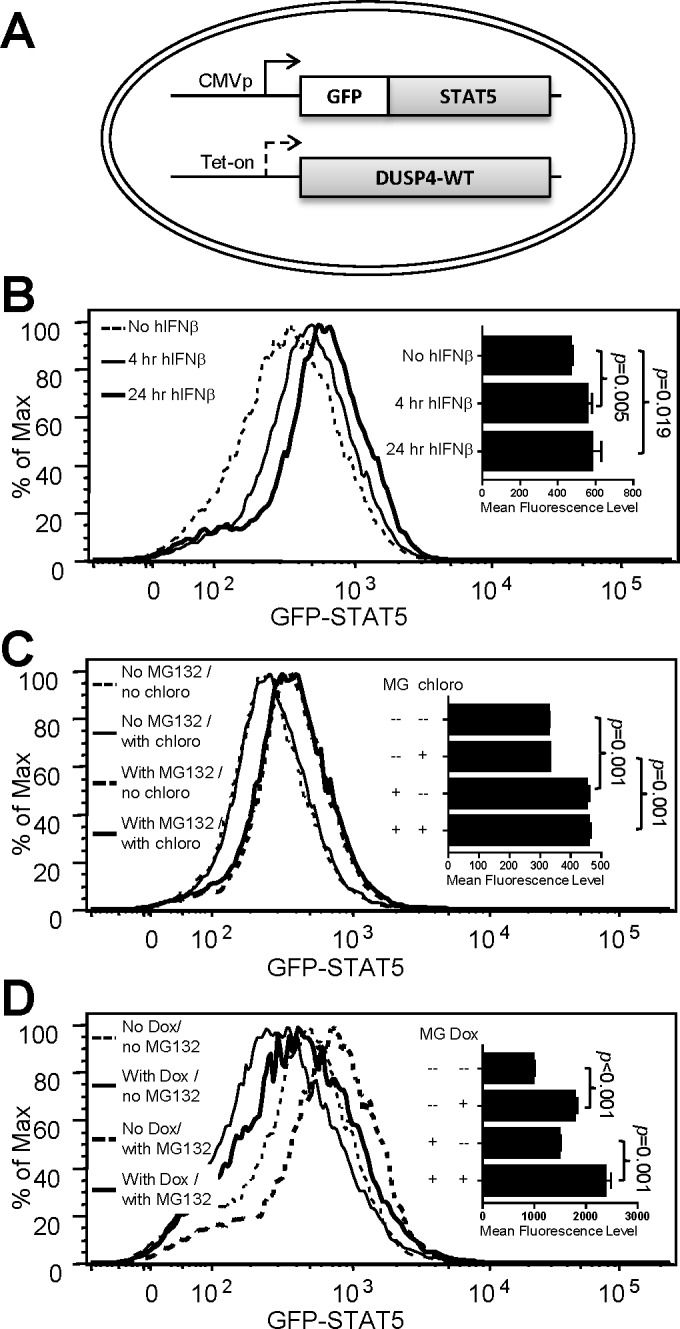
hIFNβ and MG132 increase the steady-state level of STAT5 but fail to suppress DUSP4-mediated STAT5 down-regulation. (A) Schematics of the 293-TO-D4-WT line with stable GFP-STAT5 fusion protein expression. CMVp, CMV promoter. Tet-on, tetracycline-inducible promoter. (B) The cell line in A was treated with hIFNβ for the indicated time, followed by FACS analyses for the steady-state levels of the GFP-STAT5 fusion protein. Representative histogram overlays, as well as statistical analyses results of the mean fluorescence levels (panel insert), from three experiments are shown. (C) Cells were treated and analyzed as in panel B, except with the addition of MG132 (MG) or chloroquine (chloro) treatment as indicated during the last 6 hr of culture period. Representative histogram overlays, as well as statistical analyses results of the mean fluorescence levels (panel insert), from three experiments are shown. (D) Cells were treated and analyzed as in panel B, except with the addition of doxycycline (Dox) (24hr) or MG132 (MG) (6hr) treatments as indicated. Representative histogram overlays, as well as statistical analyses results of the mean fluorescence levels (panel insert), from three experiments are shown. See also Figure C in S2 Fig.

Using 293-TO-D4-WT cell lines with stable transfection of GFP-STAT5 (Fig 7A), we then assessed the effects of hIFNβ, MG132, chloroquine and DUSP4 on the steady-state level of GFP-STAT5 *in vitro*. Human IFNβ treatment gradually increased the protein levels of GFP-STAT5 between 4 and 24 hours (*p*<0.05–0.019, Fig 7B). MG132 treatment also increased the levels of GFP-STAT5 (*p* = 0.001, Fig 7C), but chloroquine treatment appeared to have no effect (Fig 7C), suggesting that proteasome but not lysosome was involved in STAT5 protein homeostasis. Lastly, doxycycline treatment and the resulting induction of WT DUSP4 reduced the levels of GFP-STAT5 (*p*<0.001, Fig 7D, top two rows) to again support the DUSP4-mediated destabilization of STAT5. However, simultaneous treatments with MG132 failed to rescue this down-regulation (*p* = 0.001, Fig 7D, bottom two rows). These observations thus suggest that DUSP4 may modulate the protein levels of STAT5 via proteasome- and lysosome-independent pathways.

### The coiled coil domain of STAT5 negatively regulates DUSP4-STAT5 interaction and DUSP4-mediated STAT5 destabilization

**Fig 8:**
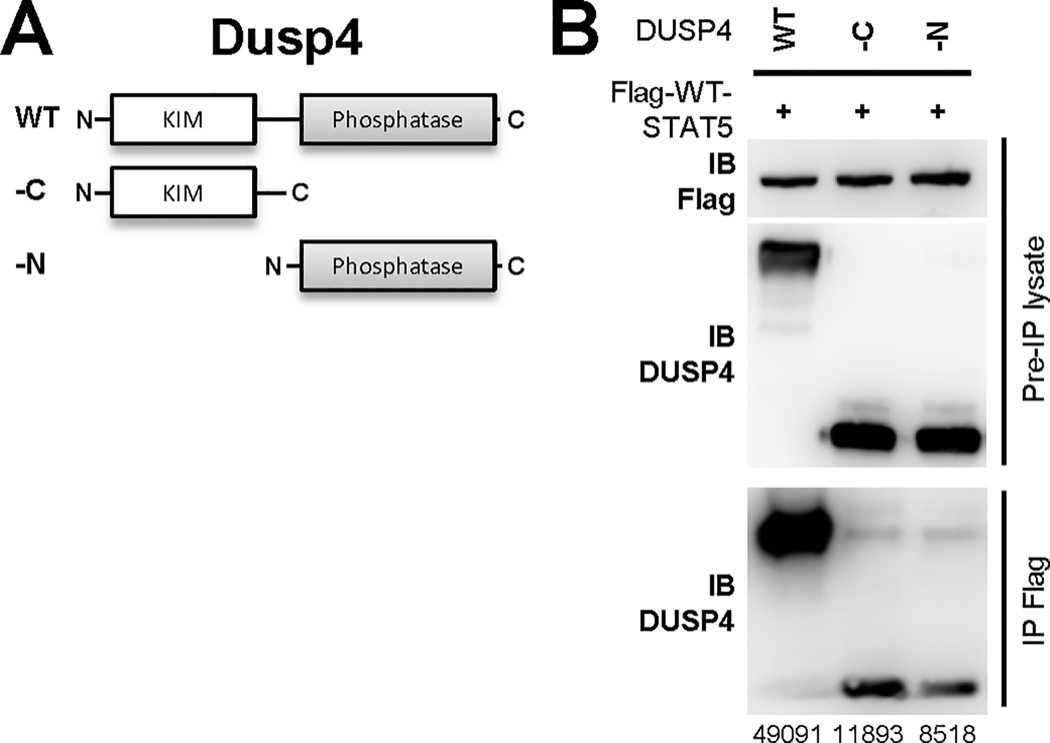
Both the KIM and phosphatase domains of DUSP4 are required for DUSP4’s optimal interaction with STAT5. (A) Schematics of DUSP4 mutants.–N, N-terminal-truncated mutant.–C, C-terminal-truncated mutant. KIM, kinase-interacting motif. (B) HEK-293T cells were co-transfected with WT or mutant DUSP4 and Flag-tagged WT STAT5 constructs for 24 hr, followed by lysate collection and anti-Flag IP. Western blotting results of the pre-IP lysates (top two rows) and the precipitated fractions (IP Flag, bottom row) are shown. The respective band intensities of DUSP4 in IP Flag are indicated. Representative results from two independent experiments are shown.

**Fig 9:**
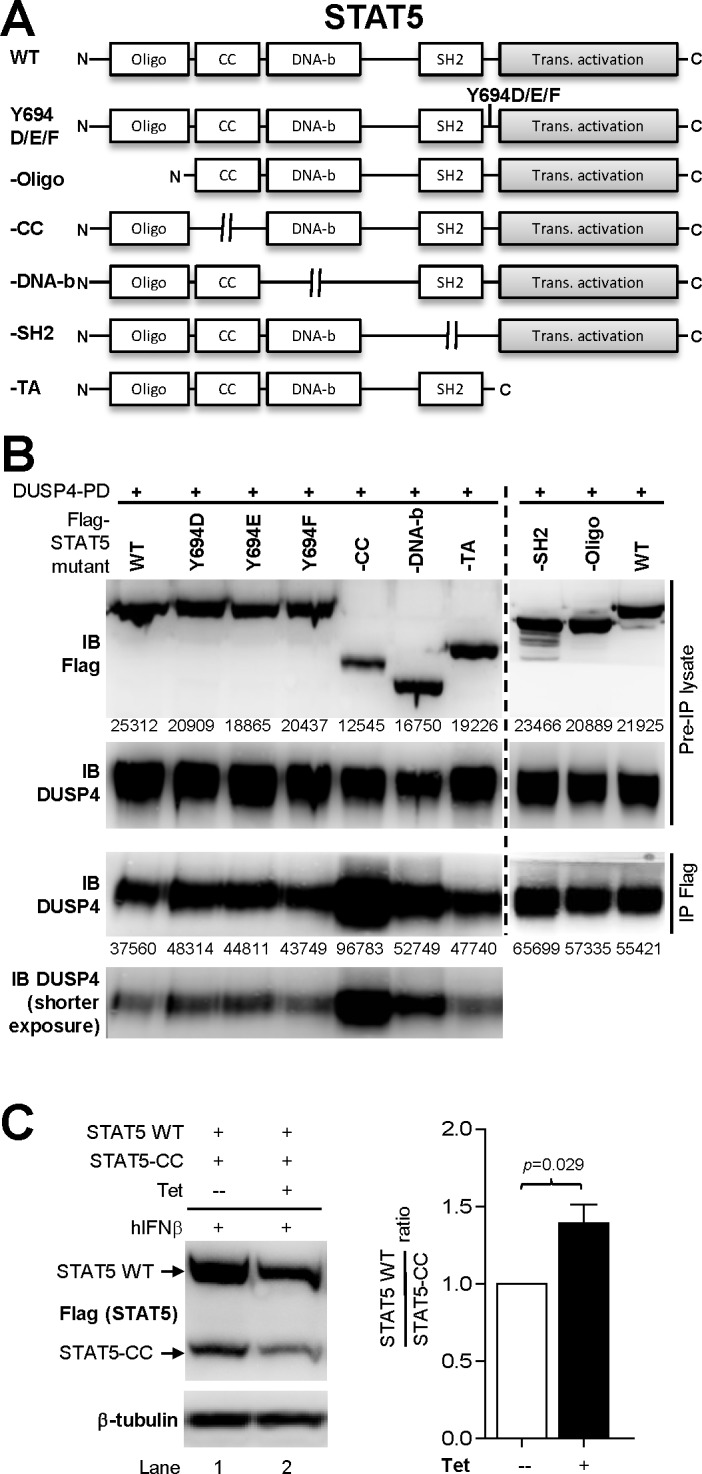
The coiled-coil domain of STAT5 negatively regulates DUPS4-STAT5 interaction strength and DUSP4-mediated STAT5 down-regulations. (A) Schematics of STAT5 mutant constructs. Oligo, oligomerization domain. CC, coiled-coil domain. DNA-b, DNA-binding domain. SH2, SH2 domain. TA/Trans. Activation, transcription activation domain. (B) HEK-293T cells were co-transfected with DUSP4-PD and various Flag-tagged STAT5 constructs for 24 hr, followed by lysate collection and anti-Flag IP. Western blotting results of the pre-IP lysates (top two rows) and the precipitated fractions (IP Flag, bottom row) are shown. The respective band intensities of flag-STAT5 in Pre-IP lysate and DUSP4 in IP Flag are indicated. A shorter exposure of the IB DUSP4 results in the IP Flag fraction is also shown. Representative results from four independent experiments are shown. See also Figure D in S2 Fig. (C) 293-TO-D4-WT clones were co-transfected with WT (STAT5-WT) and coiled-coil domain mutant STAT5 (STAT5-CC) and analyzed as in Fig 9B. Representative blotting results from three independent clones are shown (left panel), as are the ratios of WT STAT5 over coiled-coil domain mutant STAT5 normalized to the no-tetracycline control (*n* = 5, right panel). Tet, tetracycline.

We have previously demonstrated the interaction between DUSP4 and STAT5 in both primary thymocytes and HEK-293T cells [15]. To gain more mechanistical insights of this interaction, we performed domain-mapping by generating N-terminal- (-N) or C-terminal-truncated mutant DUSP4 (Fig 8A), as well as various deletion mutants of STAT5 by removing individually the oligomerization, coiled-coil, DNA-binding, SH2, and transcription activation domains; in addition, phosphomimic (Tyr to Asp/Glu) or phosphorylation-defective (Tyr to Phe) point mutants at the Y694 phosphorylation site were also generated (Fig 9A). Results from the co-IP experiments indicated that both the N-terminal KIM domain and the C-terminal phosphatase domain of DUSP4 were required for its optimal interaction with STAT5, as the removal of either domain reduced the co-IP efficacy (Fig 8B). In contrast, when tested for their ability to co-IP with DUSP4-PD (DUSP4-PD but not WT DUSP4 was used to avoid potential dephosphorylation and degradation of STAT5), none of the STAT5 deletion mutants exhibited reduced co-IP efficacy (Fig 9B), suggesting that no single STAT5 domain was essential for this STAT5-DUSP4 interaction. In addition, mutations at the Y694 residue also did not alter the strength of DUSP4-STAT5 interaction (Fig 9B), suggesting that Y694 phosphorylation of STAT5 likely does not regulate STAT5’s interaction with DUSP4.

While none of the STAT5 domain deletions abolished the co-IP of STAT5 with DUSP4, the removal of the STAT5 coiled-coil domain actually enhanced the co-IP efficacy (Fig 9B, bottom panel). Moreover, this mutation also reduced the steady-state level of STAT5 proteins in the pre-IP lysates (Fig 9B, top panel), indicating that the STAT5 coiled-coil domain mutant may be more susceptible than WT STAT5 to DUSP4-mediated down-regulations. To test this possibility, we co-transfected WT and coiled-coil domain mutant STAT5 into 293-TO-D4-WT cells, and compared their relative abundance in the presence or absence of tetracycline-induced WT DUSP4. The results showed that, while DUSP4 reduced the levels of both WT and coiled-coil mutant STAT5 (comparing lane 1 and 2, Fig 9C, left panel), band intensities quantifications revealed a ~40% increase in the ratio of WT STAT5 over coiled-coil domain mutant STAT5 upon the induction of DUSP4 (*p* = 0.029, Fig 9C, right panel), suggesting that the STAT5 mutant missing the coiled-coil domain is indeed more susceptible to the down-regulation by DUSP4. Combined with the increased co-IP efficacy using the same construct (Fig 9B), results from the coiled-coil domain mutant STAT5 provide an interesting correlation between the strength of DUSP4-STAT5 interaction and the extent of DUSP4-mediated down-regulations of STAT5, thereby implying that DUSP4 may mediate the reduction of STAT5 via protein-protein interactions.

## Discussion

In this report, we provided unambiguous genetic evidence that DUSP4 deficiency results in enhanced Treg differentiation and reduced Th17 polarization, as well as enhances the resistance to EAE inductions. In other mouse models, deficiency in DUSP4 has been linked with increased susceptibility to *Leishmania major* infections due to reduced Th1 but increased Th2 responses [14]; the deletion of DUSP4 also introduces resistance to LPS-induced sepsis by reducing the secretion of pro-inflammatory cytokines TNF, IL-1, and IL-6 [13]. In retrospect, the increased Treg numbers and the resulting anti-inflammation responses in DUSP4^-/-^ mice could potentially suppress the Th1 response and prevent the clearance of *Leishmania*; they might also inhibit the activation of innate immune cells and reduce the production of pro-inflammatory cytokines [13, 14]. It may thus be of interest to re-evaluate the involvements of altered Treg responses in these two DUSP4^-/-^ animal models, so that possible upstream roles of Treg cells can be assessed.

Although an existing report has associated DUSP4 with human Treg development [48], how DUSP4 may regulate Treg polarization or homeostasis is still unclear. We believe that data in this report provide additional mechanistic insights to DUSP4’s involvements in Treg development: when naïve T cells were stimulated with anti-CD3 and anti-CD28 without exogenous cytokines or antibodies, the basal FOXP3-GFP induction is similar between WT and DUSP4^-/-^ T cells (Fig 2B and 2C); together with our previous results showing normal TCR signaling in DUSP4^-/-^ T cells [15], these data suggest that DUSP4 likely does not regulate Treg differentiation by modulating the TCR signaling strength, a proposed mechanism for Treg differentiation in the thymus (reviewed in [49]). Similarly, while ERK2 is a potential target of DUSP4 [4, 5] and has been reported to suppress the differentiation of Treg cells [50], the unaltered ERK1/2 activation in DUSP4^-/-^ T cells [15] also argues against ERK2 as the mediator of the described Treg phenotypes. Finally, the expression levels of SOCS family genes are also unaltered by DUSP4 deficiency (Figure A in S3 Fig), and thus rules out an indirect modulation of STAT5 via SOCS proteins. In contrast, our observed effects of DUSP4 on STAT5 phosphorylation ([15] and Fig 4B), STAT5 protein stability (this report), and STAT5 transcription factor activity (this report), together with the reported functions of IL-2 [18, 19] and STAT5 [30, 51] in regulating Treg cell development, support a role of the IL-2/STAT5 signaling pathway for the altered Treg homeostasis in DUSP4^-/-^ mice. Regardless, the potential involvement of IL-2-independent mechanisms, e.g. the ability of DUSP4 to negatively feedback-regulate TGFβ signaling [12, 52], should not be excluded, as our iTreg polarization results showed that the induction of FOXP3-GFP is still slightly better in DUSP4^-/-^ cells even when IL-2 signaling are blocked by neutralizing antibodies (*p* = 0.08, Fig 2D). In summary, although the relative involvements of IL-2/STAT5 and TGFβ in the observed Treg phenotypes await further investigations, our findings in this report help provide the framework for future mechanistic studies on DUSP4’s functions over helper T cell differentiation.

To our knowledge, our report is the first to demonstrate the destabilization of STAT5 via phosphatase-dependent mechanisms. Regarding the pathways involved for this destabilization, it has been shown that MG132 can achieve an effective [43] or marginal [42] blockade of STAT5 degradation. Our data indicate that MG132 treatment can increase the steady-state level of GFP-STAT5 reporter (Fig 7C and 7D), thereby corroborating the involvement of proteasome in the regulation of STAT5 protein stability; however, the same data also clearly show that MG132 failed to prevent STAT5 reduction in the presence of DUSP4 over-expression (Fig 7D). Combined with the observation that chloroquine has no effect at all (Fig 7C), our results imply that this DUSP4-mediated STAT5 destabilization may predominantly utilize proteasome- and lysosome-independent pathways; this possibility is partly supported by an existing report showing that STAT5degradation can be mediated via proteasome-independent pathways [53]. A systematical test of inhibitors of different protein degradation pathways, including the autophagy pathway, may be necessary to uncover the underlying mechanisms.

Our analyses show that the coiled-coil domain of STAT5 negatively regulates the interaction between DUSP4 and STAT5, as the deletion of this domain enhances the efficacy of DUSP4-STAT5 co-IP (Fig 9B). Currently no co-crystal structure is available for DUSP4 and STAT5, making it difficult to surmise the reason behind this enhanced co-IP efficacy. Still, one logical assumption is that the coiled-coil domain, which is exposed on the surface irrespective of STAT5’s phosphorylation status [25, 54], could introduce steric hindrance between DUSP4 and STAT5 to mediate this effect. More importantly, the ability of the coiled coil domain to regulate DUSP4-STAT5 interaction leads us to speculate on a potential function of DUSP4 in controlling the nuclear shuttling of STAT5, as discussed below: an existing report suggests that the coiled-coil domain of STAT5 may contain an unconventional, likely conformational, motif for the nuclear import of STAT5 [26], yet the grafting of STAT5’s coiled-coil domain fails to grant nuclear import capacity to the target protein [40]. One possible explanation for these two seemingly paradoxical findings is that the coiled-coil domain of STAT5 may not directly mediate the nuclear import of STAT5 but, instead, may indirectly prevent its nuclear export via the function of an interacting partner. In this respect, our preliminary data showed that DUSP4-PD (which does not mediate STAT5 dephosphorylation or protein destabilization, but does interact strongly with STAT5) can still down-regulate the transcription factor activity of STAT5 in reporter assays (Figure B in S3 Fig), thereby implying a dephosphorylation- and destabilization-independent form of function modulation for STAT5. These observations therefore lead us to propose a model in which DUSP4-PD (and likely WT DUSP4 as well) suppresses STAT5’s transcription factor activity by binding to STAT5 and mediating its nuclear export; in addition, this DUSP4-STAT5 interaction, and the resulting DUSP4-mediated nuclear export of STAT5, is antagonized by the coiled coil domain. We are currently investigating these hypotheses, and the outcomes of these investigations may add a third dimension to the DUSP4-mediated regulations of STAT5.

Since STAT5 is involved in the signaling of many cytokines [28], one may expect multiple forms of immune dysregulations to be observed in DUSP4^-/-^ mice. However, DUSP4^-/-^ mice did not exhibit clear alteration in the OVA T cell responses or NP-specific antibody production [15]; in addition, DUSP4 deficiency has not been linked with the onset of severe autoimmune or immune-deficient syndromes. We believe that this lack of additional immune phenotypes in DUSP4^-/-^ mice, as well as the relatively slight increase of STAT5 protein levels in DUSP4^-/-^ T cells (Fig 4A), may both be attributed to potential functional redundancies between DUSP4 and related DUSPs. Coming from a fairly large family with 25 members, it is conceivable that different DUSPs may have overlapping functions, a possibility that is supported by a recent report showing that concomitant deletions of DUSP1 and DUSP4 are required for the induction of cardiomyopathy [55]. Along the same line, helper T cell development has also been shown to be controlled in parallel by different DUSPs: results from the over-expression of DUSP5 [56] or DUSP6 [57] show that these DUSPs can change thymocyte signaling thresholds to alter positive selection and the induction of autoimmune T cells; in the periphery, hydrodynamic induction of DUSP5 induces resistance to autoimmune arthritis by altering the Treg/Th17 balance *in vivo* [58]; analyses of gene-deficient mouse models further show that DUSP2 can positively regulate autoimmune arthritis [59], while DUSP10 can enhances the production of Th1/Th2 cytokines [60]. Different DUSPs may thus cooperate to modulate helper T cell homeostasis and functions. In this regard, DUSP4 may have additional roles in immune modulations that can only be revealed when multiple DUSPs are inactivated simultaneously.

## Supporting Information

S1 Fig Flow cytometry gating strategies and validation of cell sorting. (Figure A) Gating strategies for Figs 1B, 1C, 2A, 2B, 2C and 2D. (Figure B) Gating strategies for Figs 5D, 5E, 7B, 7C and 7D. (Figure C) Post-sort flow cytometry analyses of MACS-purified primary naïve CD4 T cells for Treg polarization experiments.
(PDF)

S2 Fig Western blotting and flow cytometry analysis results from independent, duplicate experiments. (Figure A) Two duplicate experiments for Fig 4A showing increased STAT5 protein levels in DUSP4^-/-^ primary T cells. The respective band intensities and the ratio of STAT5/Tubulin signals are shown. D4^-/-^, DUSP4^-/-^. Gradient acrylamide gels (4–15%) were used in the left panel, and allowed the separation of endogenous STAT5a and STAT5b as two distinguished bands. (Figure B) Two duplicate experiments for Fig 4B showing reduced STAT5 phosphorylation and protein levels in HEK-293T cells over-expressing DUSP4. The respective band intensities and the ratio of p-STAT5/STAT5 and STAT5/Tubulin signals are shown. p-STAT5, Y694 phosphorylated STAT5. (Figure C) Two duplicate experiments for Fig 7D showing reduced GFP-STAT5 mean fluorescence levels when Tet-on DUSP4 was induced. MG, MG132 treatment. Dox, doxycycline treatment. (Figure D) Two duplicate experiments for Fig 9B showing enhanced DUSP4-STAT5 co-IP efficacy by deleting the coiled coil domain (-CC). The respective band intensities for pre-IP flag-STAT5 and co-IP DUSP4 are shown. Gradient acrylamide gels (4–15%) were used in the right panel, and allowed the separation of post-translationally-modified forms of DUSP4 as two distinct bands.
(PDF)

S3 Fig Gene expression analyses of SOCS and STAT5a/b in primary T cells, and DUSP4-PD-mediated regulations on the transcription factor activity of STAT5. (Figure A) Microarray analysis results for SOCS and STAT5 mRNA levels in splenic T cells. Naïve T cells were purified by MACS as described in the Materials and Method section. mRNA was extracted with Trizol and validated by Bio-analyzer, followed by hybridization with the Mouse Gene 2.0 ST array chip at the Gene Microarray Core Facility at the NHRI using GeneChip Hybridization oven 640, GeneChip Fluidics Station 450, and GeneChip Scanner 3000. Data analyses were performed with Transcriptome Analysis Console 3.0 (Affymetrix). Fold changes for the mRNA levels of the respective genes, as well as the *p* value from ANOVA analysis, are shown. Results from 3 independent pairs of WT and DUSP4^-/-^ T cells samples are shown (http://www.ncbi.nlm.nih.gov/geo/query/acc.cgi?acc=GSE75319). (Figure B) 293-TO-D4-PD clones were transfected, treated and analyzed with luciferase reporter assays as in Fig 3B (*n* = 3).
(PDF)

S1 Table Oligonucleotide sequences, their PCR applications, annealing temperatures (Temp), and cycle numbers (Cycle #). (PDF)
