# Supplementary figures and images for: Dual-Specificity Phosphatase 4 Regulates STAT5 Protein Stability and Helper T Cell Polarization*
Source: PLoS One. 2015 Dec 28;10(12):e0145880. doi: 10.1371/journal.pone.0145880 (PMC4692422; doi:10.1371/journal.pone.0145880)

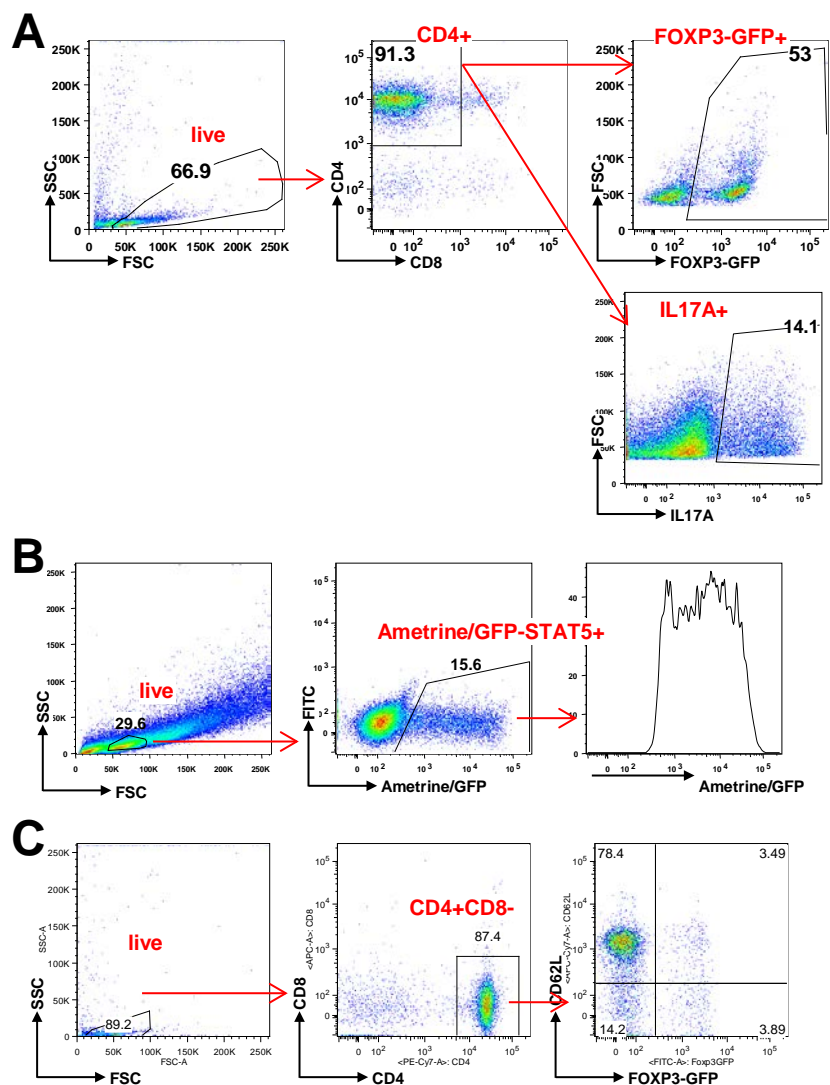

**S1 Fig. Flow cytometry gating strategies and validation of cell sorting.**

Supplement: S1 Fig — (Figure A) Gating strategies for Figs 1B, 1C, 2A, 2B, 2C and 2D. (Figure B) Gating strategies for Figs 5D, 5E, 7B, 7C and 7D. (Figure C) Post-sort flow cytometry analyses of MACS-purified primary naïve CD4 T cells for Treg polarization experiments. (PDF) [file pone.0145880.s001.pdf]
