# Supplementary material for: Dual-Specificity Phosphatase 4 Regulates STAT5 Protein Stability and Helper T Cell Polarization*
Source: PLoS One. 2015 Dec 28;10(12):e0145880. doi: 10.1371/journal.pone.0145880 (PMC4692422; doi:10.1371/journal.pone.0145880)
Supplement: S2 Fig — (Figure A) Two duplicate experiments for Fig 4A showing increased STAT5 protein levels in DUSP4-/- primary T cells. The respective band intensities and the ratio of STAT5/Tubulin signals are shown. D4-/-, DUSP4-/-. Gradient acrylamide gels (4–15%) were used in the left panel, and allowed the separation of endogenous STAT5a and STAT5b as two distinguished bands. (Figure B) Two duplicate experiments for Fig 4B showing reduced STAT5 phosphorylation and protein levels in HEK-293T cells over-expressing DUSP4. The respective band intensities and the ratio of p-STAT5/STAT5 and STAT5/Tubulin signals are shown. p-STAT5, Y694 phosphorylated STAT5. (Figure C) Two duplicate experiments for Fig 7D showing reduced GFP-STAT5 mean fluorescence levels when Tet-on DUSP4 was induced. MG, MG132 treatment. Dox, doxycycline treatment. (Figure D) Two duplicate experiments for Fig 9B showing enhanced DUSP4-STAT5 co-IP efficacy by deleting the coiled coil domain (-CC). The respective band intensities for pre-IP flag-STAT5 and co-IP DUSP4 are shown. Gradient acrylamide gels (4–15%) were used in the right panel, and allowed the separation of post-translationally-modified forms of DUSP4 as two distinct bands. (PDF) [file pone.0145880.s002.pdf]

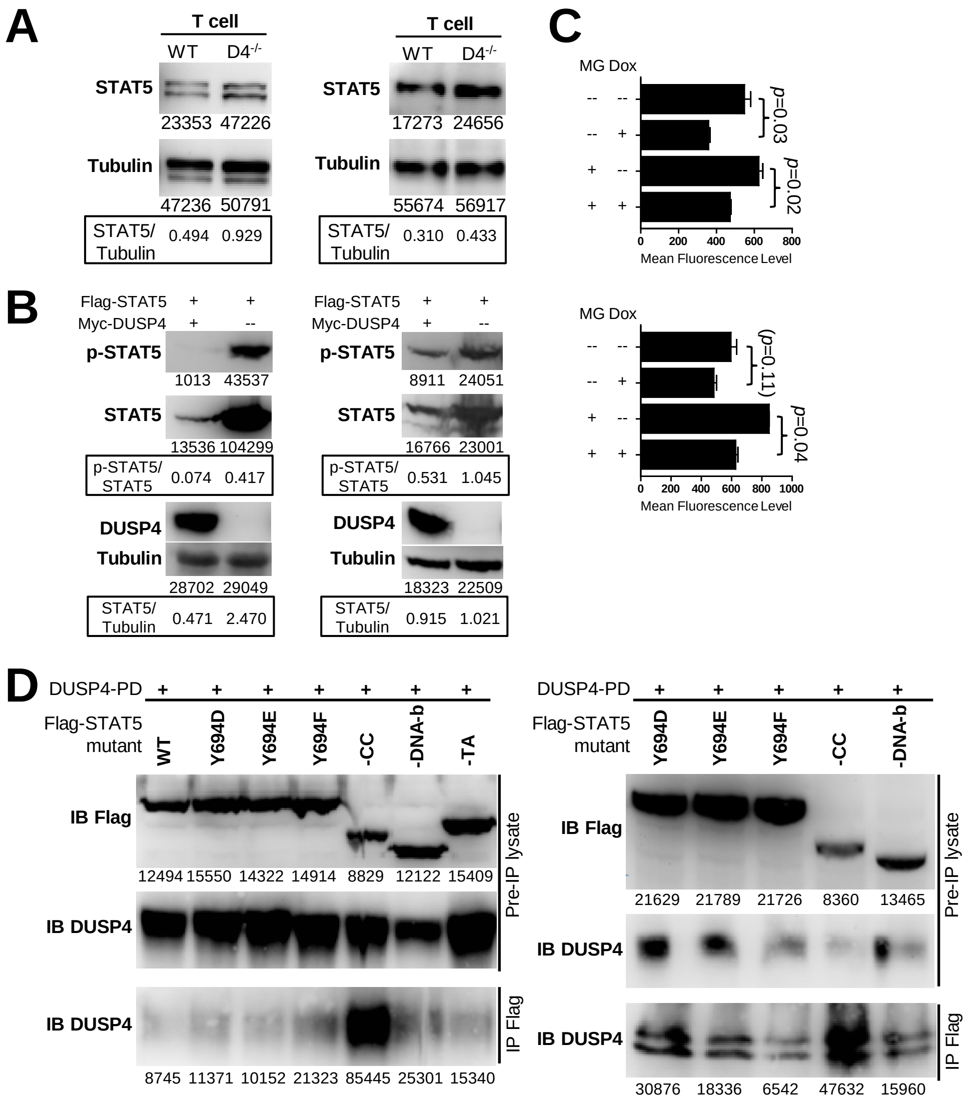

S2 Fig. Western blotting and flow cytometry analysis results from independent, duplicate experiments.

S2 Fig
