# Supplementary material for: Dual-Specificity Phosphatase 4 Regulates STAT5 Protein Stability and Helper T Cell Polarization*
Source: PLoS One. 2015 Dec 28;10(12):e0145880. doi: 10.1371/journal.pone.0145880 (PMC4692422; doi:10.1371/journal.pone.0145880)
Supplement: S3 Fig — (Figure A) Microarray analysis results for SOCS and STAT5 mRNA levels in splenic T cells. Naïve T cells were purified by MACS as described in the Materials and Method section. mRNA was extracted with Trizol and validated by Bio-analyzer, followed by hybridization with the Mouse Gene 2.0 ST array chip at the Gene Microarray Core Facility at the NHRI using GeneChip Hybridization oven 640, GeneChip Fluidics Station 450, and GeneChip Scanner 3000. Data analyses were performed with Transcriptome Analysis Console 3.0 (Affymetrix). Fold changes for the mRNA levels of the respective genes, as well as the p value from ANOVA analysis, are shown. Results from 3 independent pairs of WT and DUSP4-/- T cells samples are shown (http://www.ncbi.nlm.nih.gov/geo/query/acc.cgi?acc=GSE75319). (Figure B) 293-TO-D4-PD clones were transfected, treated and analyzed with luciferase reporter assays as in Fig 3B (n = 3). (PDF) [file pone.0145880.s003.pdf]

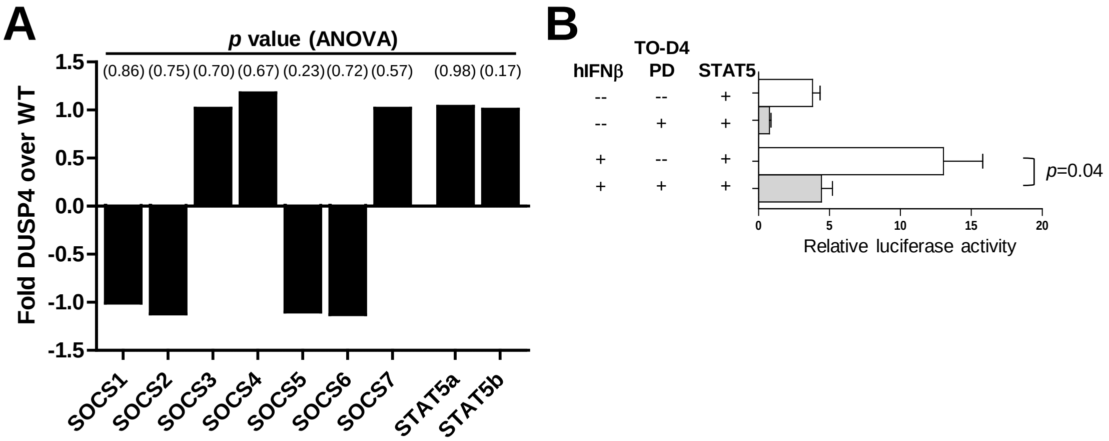

S3 Fig. Gene expression analyses of SOCS and STAT5a/b in primary T cells, and DUSP4-PD-mediated regulations on the transcription factor activity of STAT5.

S3 Fig
