# Supplementary material for: Dual-Specificity Phosphatase 4 Regulates STAT5 Protein Stability and Helper T Cell Polarization*
Source: PLoS One. 2015 Dec 28;10(12):e0145880. doi: 10.1371/journal.pone.0145880 (PMC4692422; doi:10.1371/journal.pone.0145880)
Supplement: S1 Table — (PDF) [file pone.0145880.s004.pdf]

S4 Table. Oligonucleotide sequences, their PCR applications, annealing temperatures (Temp), and cycle numbers (Cycle #).

Name2	5'-3' sequences	Application	Temp	Cycle #
FOXP3-2, 5'	GTGCTGGACATGTGCATGTGTAGAA	FOXP3-GFP Genotyping	62 ˚C	28
FOXP3-2, 3'	TTCCAGCTTGGCAAGACTCCTG	FOXP3-GFP Genotyping	62 ˚C	28
eGFP-3, 3'	AGCTTGTGCCCCAGGATGTTG	FOXP3-GFP Genotyping	62 ˚C	28
XG164-13,5'	ACCAACCTTGGGATAGGGGAGAGA	DUSP4 Genotyping	62 ˚C	28
XG164-3,5'	TTTAGGAGCGACGGCCAGGA	DUSP4 Genotyping	62 ˚C	28
KR-3	GGAAAGGGTAAAGTGGTAGGG	DUSP4 Genotyping	62 ˚C	28
DUSP4, 5'	GATGGCAACTTCAACTGGGGC	qPCR	55 ˚C	40
DUSP4, 3'	TTGGATCCAGACAAGCAGCCG	qPCR	55 ˚C	40
STAT5-8.5'	ACAGCATGTCTGTGTCCTGGTC	qPCR	55 ˚C	40
STAT5-8.3'	GTCCCGTCCGGCTTGTTGAT	qPCR	55 ˚C	40
Actin, 5'	AAGTGTGACGTTGACATCCGTAA	qPCR	55 ˚C	40
Actin, 3'	TGCCTGGGTACATGGTGGTA	qPCR	55 ˚C	40
mD4-5,5'+AsisI	GAGGCGATCGCATGGTGACGATGGAGGAAC	General DUSP4 cloning	60 ˚C	32
mD4-5,3'+MluI	GCGACGCGTACAGCTGGGGGAGGTGGT	General DUSP4 cloning	60 ˚C	32
mD4-8, 3'+MluI	GCGACGCGTGGACTCATTGGTGCTGGGAGG	-C mutant DUSP4 cloning	60 ˚C	32
mD4-9, 5'+AsiSI+C+ATG	GAGGCGATCGCCATGCCTCCCAGCACCAATGAGTCC	-N mutant DUSP4 cloning	60 ˚C	32
mD4-4, 5'	CTGGTTCACAGCCAGGCCGG	C284S mutant DUSP4-PD cloning	60 ˚C	32
STAT5-1, '+AsiSI	GAGGCGATCGCCATGGCGGGCTGGATTCAGG	General STAT5 cloning	60 ˚C	32
STAT5-1, 3'+MluI	GCGACGCGTGGACAGGGAGCTTCTAGCGG	General STAT5 cloning	60 ˚C	32
STAT5-2, 5'+AsiSI+Met	GATGGCAACTTCAACTGGGGC	-oligo mutant STAT5 cloning	60 ˚C	30
STAT5-3, 5'+OL	GACGCCATCATCTCAGCTCTGGTCACCAGC	-CC mutant STAT5 cloning	60 ˚C	30
STAT5-2, 3'+OL	GATGATGGCGTCAACCAGGACACC	-CC mutant STAT5 cloning	60 ˚C	30
STAT5-4, 5'+OL	ACCAGCTGGTTCGACGGGGTGATGGAG	-DNA-b mutant STAT5 cloning	60 ˚C	30
STAT5-3, 3'+OL	GAACCAGCTGGTGACCAGAGCTGAGATGAT	-DNA-b mutant STAT5 cloning	60 ˚C	30
STAT5-5, 5'+EcoRV	CGGATATCTCCACAGATGCCGGAGCC	-SH2 mutant STAT5 cloning	62 ˚C	30
STAT5-4, 3'+EcoRV	CGGATATCCTCCATCACCCCGTCGAACCA	-SH2 mutant STAT5 cloning	62 ˚C	30
STAT5-5, 3'+MluI	GCGACGCGTGGCTCCGGCATCTGTGGA	-TA mutant STAT5 cloning	60 ˚C	30
STAT5-Y694D-1, 5'	GTTGACGGAGACGTGAAGCCACAG	Y694D mutant STAT5 cloning	60 ˚C	30
STAT5-Y694D-1, 3'	CTGTGGCTTCACGTCTCCGTCAAC	Y694D mutant STAT5 cloning	60 ˚C	30
STAT5-Y694E-1, 5'	GTTGACGGAGAAGTGAAGCCACAG	Y694E mutant STAT5 cloning	60 ˚C	30
STAT5-Y694E-1, 3'	CTGTGGCTTCACTTCTCCGTCAAC	Y694E mutant STAT5 cloning	60 ˚C	30
STAT5-Y694F-1, 5'	GTTGACGGATTCGTGAAGCCACAG	Y694F mutant STAT5 cloning	60 ˚C	30
STAT5-Y694F-1, 3'	CTGTGGCTTCACGAATCCGTCAAC	Y694F mutant STAT5 cloning	60 ˚C	30
Ametrine-1, 5'+AsiSI	GAGGCGATCGCCATGGTGAGCAAGGGCGAGG	Ametrine cloning	62 ˚C	32
Ametrine-1, 3'+AsiSIx5	GTGAGGCGATCGC+GCCGAGAGTGATCCCGGC	Ametrine cloning	62 ˚C	32
eGFP_1-5'+AsiSI	GCCGCGCGATCGCATGGTGAGCAAGGGCGAG	GFP cloning	62 ˚C	32
eGFP_1-3'+AsiSI	TCGCGGCGATCGCCTTGTACAGCTCGTCCAT	GFP cloning	62 ˚C	32
Ametrine-1, 5'+AsiSI	GAGGCGATCGCCATGGTGAGCAAGGGCGAGG	tdTomato cloning	62 ˚C	32
tdTomato-1, 3'+RsrII	GCGTCGGTCCGCTCTTGTACAGCTCGTCCATGCCGTA	tdTomato cloning	62 ˚C	32

S4 Table
